# Emotional support predicts more sickness absence and poorer self assessed work ability: a two-year prospective cohort study

**DOI:** 10.1186/1471-2458-10-648

**Published:** 2010-10-26

**Authors:** Nadine Karlsson, Elisabeth Skargren, Margareta Kristenson

**Affiliations:** 1 Department of Medical and Health Sciences, Division of Community Medicine, Social Medicine and Public Health Science, Linköping University, Linköping, Sweden; 2 Department of Medical and Health Sciences, Division of Physiotherapy, Linköping University, Linköping, Sweden

## Abstract

**Background:**

While back pain and stressful work environment are shown to be important causes of sickness absence the effect of psychosocial resources on sickness absence, and on self assessed work ability, is less commonly investigated. The aim of this study was to assess these associations in a two-year follow-up study.

**Methods:**

341 working people aged 45 to 64, randomly drawn from the population, responded to a questionnaire at baseline and at a two-year follow-up. Poisson regression was used to analyse the association of psychosocial factors (psychosocial instruments on work environment, emotional support and psychological resources) and previous back pain (low back and/or neck) at baseline with sickness absence (spells and days) at follow-up, controlling for effects of age, sex, BMI, smoking, alcohol, occupation, disease and previous sickness absence. Logistic regression was used to study the associations of psychosocial factors and previous back pain at baseline with self assessed prognosis of poor work ability six months from follow-up. Finally, a multivariate analysis tested the independent effects of previous back pain and 3 psychosocial factors derived in a factor analysis: 1. work environment; 2. emotional support; 3. psychological resources, on work ability and absence days and spells.

**Results:**

80% of the sickness absence spells within the last 12 months before follow-up were short-term (≤ 14 days). In the final model, high emotional support predicted *more *sickness absence spells (RR 1.36; 1.11-1.67) and days (RR 1.68, 1.22-2.31). Previous back pain (OR 2.56; 1.13-5.81), high emotional support (OR 1.58; 1.02-2.46), and low psychological resources (OR 0.62; 0.44-0.89) were related to *poorer *self assessed prognosis of work ability at follow up.

**Conclusions:**

In a general middle aged working population high emotional support was related to more sickness absence and also poorer self assessed prognosis of work ability. Our findings suggest that both sickness absence and self assessed work ability are dependent of life outside work and can be affected by a person's close community.

## Background

Musculoskeletal disorders, primarily back pain, have been the main causes for sickness absence in Sweden [1,2], but stress-related ill health is growing rapidly as a cause of such absence [3]. Measures of stressors, in terms of psychosocial working environment, predict the occurrence of cardiovascular [4], common mental disorders [5-7], but also musculoskeletal disorders [8,9], and have also been found to predict sickness absence in several prospective studies [10-16]. These studies, predominantly based on the demand-control-support model [17], have consistently found that low decision latitude is related to a high level of sickness absence [18] but there is no clear evidence for the effects of psychological demands or social support at work on such absence [19].

The examination of a wide-range of defined theory-based psychosocial characteristics is unusual in sickness absence research [20], and few studies have examined the association between psychosocial resources and sickness absence. An active problem-solving coping style has been shown to reduce the risk of sickness absence [21]. In the Whitehall II cohort, high levels of confiding/emotional support at home was associated with a higher risk for sickness absence [22] and sickness absence, mainly the shorter, has been suggested as a coping behavior to reduce work-related stress by avoiding the workplace and thereby creating the opportunity for recuperation [23].

Whereas absence due to illness could bee seen as the negative pole in the continuum between excellent and ill health, work ability is defined as the capacity of an individual to manage gainful employment as a means of earning a living. The determinants of sickness absence and work ability are not necessarily the same [24], and few investigations have examined predictors for both sickness absence and work ability in parallel.

### Study aim

The aim of this study was to investigate prospectively, over a two-year follow-up in a general middle-aged working Swedish population, the effect of psychosocial work environment, psychosocial resources and previous back pain on self-reported sickness absence and self assessed prognosis of work ability.

## Methods

### Study design and population

The Life Conditions, Stress, and Health (LSH) study [25-28] is a longitudinal study of socioeconomic differences in health in a population of men and women (non-patients) drawn from the county of Östergötland in the southeast of Sweden. It was based on a random sample of 1007 men and women between 45-69 years of age in 2003, as stratified by sex and age, who belonged to any of the catchment areas of ten primary health care centres in the region (response rate 62%). Data collection at baseline (2003 to 2004) and at follow-up (2005 to 2006) included data self-reported by postal questionnaire. Exclusion criteria were serious disease and difficulties in understanding the language. Follow-up data were collected from a total of 795 men and women (response rate 79%).

The eligible individuals for the present study were individuals who were employed both at baseline and follow-up. At baseline, 534 of the respondents in the survey were employed. 33 respondents were sick-listed at baseline and therefore excluded. Of the 501 baseline respondents, 409 returned the follow-up questionnaire (82% response rate). Another 68 individuals were no longer employed at the time of the follow-up, resulting in a final study sample of 341 individuals (prospective cohort). At follow-up the proportion of individuals with a manual occupation was higher in non respondents than in respondents (p = 0.002).

### Measurements

#### Self-reported sickness absence

The self-reported number of sickness absence spells (or periods) and days were measured at baseline and at two-year follow-up by the question: "How many spells of sickness absence did you have in the last 12 months?" and "How many days of sickness absence did you have in the last 12 months?"

Measuring both the number of sickness absence spells and their duration is important, since they are only partly determined by the same variables. For example, sickness absence frequency is more influenced by an employee's task, workgroup organisation, leadership, shift work, and absence control measures, whereas sickness absence duration is more influenced by age, working conditions, sickness benefits, and access to medical care and specialists [29].

#### Self assessed prognosis of work ability

Information on self assessed prognosis of work ability six months from follow-up was obtained through a question posed in previous research [30]: "How much chance is there that you will be able to work within six months?" The six response categories ranged from "very unlikely" to "very likely". The answers were dichotomised, with "very likely" representing "good work ability", and the remainder categorised as "poor work ability".

#### Psychosocial factors

Several psychosocial factors, widely used in stress research, were measured at baseline.

*Psychosocial work environment *was assessed by means of three instruments: Demand-control [31], social support at work [17], and overcommitment (specific cognitive and motivational pattern of coping with demands characterized by excessive work-related commitment) [32]. Two subscales were derived from the demand-control model: psychological demands (i.e., time pressure, and conflicting demands), and decision latitude (influence over what to do and how and when to do it). Job strain was calculated as the unweighted ratio between psychological demands and decision latitude.

Psychosocial resources were assessed in two domains: social support and psychological (individual) resources.

*Social support *was measured in terms of emotional support, which describes the availability of affectionally close and deeper emotional relationships, and was measured by means of the psychometric instrument "Availability of Attachment" [33]. An important component of emotional support is related to self-appraisal, providing support that boosts self-esteem and encourages positive self-appraisal [34]. Emotional support differs from practical support that is manifest under many forms, including practical help and financial support. Some typical examples of items in "emotional support" question are: "Do you have a confident from whom you feel you really can get support?"; "Do you have a confident who can share the emotions with you when you are happy?"

*Psychological (individual) resources *were measured with four scales. 1. Sense of coherence [35] includes three dimensions: a) the (cognitive) ability to define life events as less stressful (comprehensibility), b) mobilising resources to deal with stressors that one encounters (manageability), and c) motivation, desire, and commitment to cope (meaningfulness). 2. Mastery [36] is often used as an equivalent to coping ability and addresses the extent to which one regards the direction of one's life as being under one's own control, in contrast to being fatalistically ruled, while 3. self-esteem [36] refers to the positiveness of one's attitude towards oneself. The instrument 4. perceived control [37] measures how much an individual perceives that he/she can intentionally produce desired outcomes and prevent undesirable ones.

#### Self-reported previous low back and/or neck pain

This item was assessed at baseline through the question, "Have you had low back and/or neck pain during previous 5 years?" (yes/no)" [38].

#### Covariates

As covariates we included age, sex, socioeconomic status (SES), smoking (current smoker yes/no), alcohol consumption, body mass index (BMI), self-reporting a disease diagnosed by a physician (yes/no), and past sickness absence. They were assessed at baseline in the self-administered questionnaire of 2003-2004.

Socioeconomic status (SES) was based on an individual's occupation, measured according to the Swedish SEI coding system [39]. It distinguishes between manual workers (skilled or unskilled), non-manual workers (including administrators, professionals, and routine non-manual workers), and self-employed individuals (including farmers).

Alcohol consumption was categorized in three groups based on the weekly intake of alcohol in grams: low (< 80 g/week for women, < 110 g/week for men), medium (between 80-139 g/week for women, 110-169 g/week for men), and high intake (≥ 140 g/week for women, ≥ 170 g/week for men).

Past sickness absence was defined as the number of sickness absence spells and days the 12 months before baseline for the analysis of sickness absence, and as sickness absence (yes/no) in the 12 months before follow-up for the analysis of work ability.

### Statistical analysis

The reliability of the psychosocial scales was estimated by Cronbach's α internal consistency coefficient. The prospective impact of each psychosocial instrument and previous back pain at baseline was analysed with the number of sickness absence spells or days at follow-up using a Poisson regression model with a scale parameter to account for over-dispersion [40]. The analyses were adjusted successively for the following variables: 1) Model I for age and sex; 2) Model II adding lifestyle factors (BMI, smoking, and alcohol consumption), SES, and disease; 3) Model III all factors in the previous steps and adding past sickness absence. The prospective association between each psychosocial factor and previous back pain at baseline and a work ability rating of 'poor' at follow-up was analysed using a logistic regression in Models I to III described above.

A varimax-rotated principal components factor analysis [41] was performed among psychosocial factors (job strain, overcommitment, social support at work, emotional support, self-esteem, coping, sense of coherence, and perceived control) in order to derive composite dimensions of psychosocial factors, and thereby reduce the risk of reporting statistical significance due to multiple testing [42]. Finally the independent effect of three factors, derived from the factor analysis of all psychosocial instruments: work environment, emotional support and psychological resources as well as previous back pain, were analysed, simultaneously, as determinants of sickness absence (or, as the case may be poor work ability) with multivariate Poisson (or logistic) regression fully adjusted for covariates as above.

The correlation between the number of sickness absence spells and days, both at baseline and follow-up, was estimated by Spearman correlation coefficient. A significance level of 5% was considered to be statistically significant. SPSS 17 was used for factor analysis and SAS 9.1 for Poisson and logistic regressions.

The data had zero to 29 missing values for each variable, with the exception of self assessed work ability, which had 63 missing values (around 20% of participants). To retain all participants, imputed values were generated for the missing data from self assessed work ability. The 63 non responders for work ability had less sickness absence, for both spells and days, than the 278 respondents (all *p *< 0.01). Non response on self assessed work ability was coded as good work ability and statistical analyses were conducted using the imputed dataset [43]. A complete case statistical analysis of work ability was performed, and gave similar results to that using imputed values reported in the tables.

#### Ethical aspects

The study was approved by the Regional Committee for Research Ethics at Linköping University (ethical file number 02-0324).

## Results

### Characteristics of the study population

Descriptive statistics for the prospective cohort are provided in Table 1. Mean number of sickness absence spells and days at baseline and follow-up differed little over the two-year study (Table 1). The Spearman correlation coefficient between sickness absence at baseline and follow-up was 0.41 for number of spells, and 0.38 for number of days.

**Table 1:** Characteristics of the prospective cohort (n = 341)

Variable	Total (n)	Frequency (%)	Mean (95% CI)
Sex	341	.	.
Women	.	153 (44.9%)	.
Men	.	188 (55.1%)	.
Age (in years)	341	.	52.35 (51.84-52.86)
Occupation (SES)	337	.	.
Non manual	.	185 (54.9%)	.
Manual	.	112 (33.2%)	.
Self-employed	.	40 (11.9%)	.
Smoking, current	332	69 (20.8%)	.
BMI (kg/m^2^)	340	.	26.11 (25.70-26.52)
Alcohol consumption	325	.	.
Low^a^	.	264 (81.2%)	.
Medium^b^	.	36 (11.1%)	.
High^c^	.	25 (7.7%)	.
Disease (self report of diagnosis)	341	171 (50.2%)	.
Previous back pain	330	219 (66.4%)	.
"Poor" work ability	278	56 (20.1%)	.
Sickness absence spells at baseline†	341	.	0.47 (0.39-0.55)
Sickness absence spells at follow-up‡	340	.	0.49 (0.42-0.56)
Sickness absence days at baseline†	335	.	6.59 (4.83-8.35)
Sickness absence days at follow-up‡	340	.	6.96 (5.09-8.83)

CI = confidence interval.

^a^Low (< 80 g/week for women, < 110 g/week for men)

^b^Medium (between 80-139 g/week for women, 110-169 g/week for men)

^c^High (≥ 140 g/week for women, ≥ 170 g/week for men)

† within the last 12 months before baseline

‡ within the last 12 months before follow-up

Most of the sick leave spells were short-term as 87/121 (72%) of the individuals sick-listed within the last 12 months before baseline, and 114/142 (80%) of the individuals sick-listed within the last 12 months before follow-up, had a total of 1-14 sick leave days.

The descriptive statistics for the different psychosocial factors are presented in Table 2.

**Table 2:** Descriptive statistics of psychosocial scales and factor loadings for three dimensions of psychosocial scales.

	Descriptive statistics				Factor loadings^b^		
		
	α	n	Mean	SD	I	II	III
% variance explained					19.4	13.2	31.6

Psychosocial scales, No. of items (score range)							

I Work environment							
Job strain	.	324	0.70	0.17	0.69	.	.
Psychological demands^a^, 5 (5-20)	0.69	327	13.11	2.51	.	.	.
Decision latitude^a^, 6 (6-24)	0.63	338	18.96	2.58	.	.	.
Social support at work, 6 (6-24)	0.82	319	18.16	2.06	- 0.64	.	.
Overcommitment, 6 (6-24)	0.83	330	13.17	4.26	0.76	.	.
							
II Emotional support							
Emotional support, 6 (0-6)	0.79	338	5.52	1.13	.	0.94	.
							
III Psychological resources							
1. Sense of coherence, 13 (13-91)	0.80	338	69.00	9.28	.	.	0.78
2. Mastery, 7 (7-28)	0.75	331	23.33	3.05	.	.	0.83
3. Self-esteem, 10 (10-40)	0.87	329	32.96	4.60	.	.	0.84
4. Perceived control, 11 (11-66)	0.68	312	53.00	7.05	.	.	0.64

Note: α = Cronbach's α; SD = standard deviation.

^a^The derived subscales of job strain (psychological demands and decision latitude) are described statistically but were not included in the factor analysis.

^b^Factor loadings < 0.5 are not reported.

Three factors of psychosocial scales were identified in the factor analysis: I. work environment (including job strain, overcommitment, social support at work), II. emotional support and III. psychological resources (including 1. sense of coherence, 2. mastery, 3. self-esteem, and 4. perceived control). Together, the three derived components captured 64.2% of the total variance.

### Prospective impact of psychosocial factors and previous back pain on self-reported sickness absence

In model I adjusted for age and sex, several factors emerged as predictors of more sickness absence spells (Table 3) and days (Table 4): occupation (non manual vs. self-employed), alcohol consumption (low vs. medium intake), disease, past sickness absence, high job strain, high emotional support, and low self-esteem. Previous back pain and low decision latitude predicted more sickness absence days but not spells.

**Table 3:** Prospective impact of psychosocial characteristics and previous back pain at baseline on sickness absence spells.

Characteristic	Age and sex adjusted **(Model I)**^**†**^			Adjusted for the final model **(Model III)**^**‡**^		
		
	n	RR	95% CI	n	RR	95% CI
Occupation (SES)						
Non-manual	184	1 [ref]	.	.	.	.
Manual	112	1.18	0.89-1.57	.	.	.
Self-employed	40	*0.28*	*0.12-0.62*	.	.	.
Smoking, current	331	0.79	0.55-1.15	.	.	.
BMI	339	1.01	0.97-1.05	.	.	.
Alcohol consumption						
Low^a^	263	1 [ref]				
Medium^b^	36	*0.41*	*0.21-0.83*	.	.	.
High^c^	25	0.65	0.34-1.26	.	.	.
Disease (self report diagnosis)	340	*1.38*	*1.04-1.83*	.	.	.
Past sickness absence	340	*1.36*	*1.21-1.51*	.	.	.
I Work environment						
Job strain	323	*2.28*	*1.01-5.15*	298	1.50	0.67-3.40
Psychological demands	326	1.02	0.96-1.08	299	1.03	0.97-1.09
Decision latitude	337	0.95	0.90-1.00	309	1.00	0.95-1.07
Social support at work	318	0.95	0.88-1.01	289	0.96	0.89-1.02
Overcommitment	329	1.00	0.97-1.03	300	1.02	0.98-1.05
II Emotional support	337	*1.42*	*1.14-1.77*	307	*1.41*	*1.12-1.76*
III Psychological resources						
1. Sense of coherence	337	0.99	0.97-1.00	308	1.00	0.98-1.01
2. Mastery	330	0.96	0.92-1.01	300	0.99	0.94-1.04
3. Self-esteem	328	*0.96*	*0.93-0.99*	299	0.98	0.95-1.01
4. Perceived control	311	0.99	0.97-1.01	285	1.00	0.98-1.03
Previous back pain	329	0.94	0.69-1.28	301	0.83	0.61-1.13

The rate ratio in italics denotes significance (*p *< 0.05). (Result from Model II, see text). In model III rate ratios for confounders are not reported as separate regressions were run for each of the 10 psychosocial variables and previous back pain.

n = number of observations used; RR = rate ratio; CI = confidence interval.

^a^Low (< 80 g/week for women, < 110 g/week for men)

^b^Medium (between 80-139 g/week for women, 110-169 g/week for men)

^c^High (≥ 140 g/week for women, ≥ 170 g/week for men)

^†^Adjusted for age, sex; ^‡ ^age, sex, BMI, smoking, alcohol consumption, SES, disease (self report of diagnosis), and past sickness absence.

**Table 4:** Prospective impact of psychosocial characteristics and previous back pain at baseline on sickness absence days.

Characteristic	Age and sex adjusted **(Model I)**^**†**^			Adjusted for the final model **(Model III)**^**‡**^		
		
	n	RR	95% CI	n	RR	95% CI
Occupation (SES)						
Non-manual	184	1 [ref]	.	.	.	.
Manual	112	1.43	0.98-2.07	.	.	.
Self-employed	40	*0.32*	*0.12-0.89*	.	.	.
Smoking, current	331	1.14	0.74-1.78	.	.	.
BMI	339	1.03	0.98-1.07	.	.	.
Alcohol consumption						
Low^a^	263	1 [ref]	.	.	.	.
Medium^b^	36	*0.18*	*0.05-0.64*	.	.	.
High^c^	25	0.80	0.37-1.71	.	.	.
Disease (self report diagnosis)	340	*1.83*	*1.25-2.67*	.	.	.
Past sickness absence	334	*1.01*	*1.01-1.02*	.	.	.
I Work environment						
Job strain	323	*3.09*	*1.05-9.07*	293	1.16	0.38-3.53
Psychological demands	326	1.00	0.93-1.07	294	0.97	0.90-1.05
Decision latitude	337	*0.91*	*0.85-0.98*	303	0.97	0.90-1.05
Social support at work	318	0.93	0.85-1.02	283	0.97	0.88-1.07
Overcommitment	329	0.99	0.95-1.04	294	0.98	0.93-1.02
II Emotional support	337	*1.51*	*1.11-2.05*	301	*1.74*	*1.21-2.51*
III Psychological resources						
1. Sense of coherence	337	0.99	0.97-1.01	302	1.01	0.99-1.04
2. Mastery	330	1.00	0.94-1.06	294	1.07	1.00-1.15
3. Self-esteem	328	*0.94*	*0.90-0.97*	293	0.98	0.94-1.02
4. Perceived control	311	1.02	0.99-1.05	280	*1.05*	*1.02-1.09*
Previous back pain	329	*1.74*	*1.11-2.72*	295	1.43	0.92-2.22

The rate ratio in italics denotes significance (*p *< 0.05). (Result from Model II, see text). In model III rate ratios for confounders are not reported as separate regressions were run for each of the 10 psychosocial variables and previous back pain.

n = number of observations used; RR = rate ratio; CI = confidence interval.

^a^Low (< 80 g/week for women, < 110 g/week for men)

^b^Medium (between 80-139 g/week for women, 110-169 g/week for men)

^c^High (≥ 140 g/week for women, ≥ 170 g/week for men)

^†^Adjusted for age, sex; ^‡^Adjusted for age, sex, BMI, smoking, alcohol consumption, SES, disease (self report of diagnosis), and past sickness absence.

In Model II, adjusted also for effects of demography, lifestyle factors and disease, high emotional support was the only significant predictor of more sickness absence spells and days (data not shown) and high perceived control predicted an increased number of sickness absence days. In Model III, adjusting also for previous sickness absence caused little change in most estimates.

The multivariate analysis of previous back pain and derived components of factor analysis confirmed that high levels of emotional support were related to a significant increased number of sick-leave spells (RR = 1.36; 95% CI = 1.11-1.67; *p *= 0.004) and days (RR = 1.68; 95% CI = 1.22-2.31; *p *= 0.002) at follow-up (Table 5).

**Table 5:** Multivariate analysis of sickness absence spells and days and poor self assessed work ability.

Variables	Sick-leave spells^b^	Sick-leave days^b^	Poor work ability^c^
	**RR^a ^(95% CI)**	**RR^a ^(95% CI)**	**OR^a ^(95% CI)**

I Work environment	1.09 (0.95-1.25)	1.00 (0.84-1.19)	1.39 (0.98-1.98)
II Emotional support	1.36 (1.11-1.67)**	1.68 (1.22-2.31)**	1.58 (1.02-2.46)*
III Psychological resources	0.96 (0.82-1.11)	1.15 (0.94-1.41)	0.62 (0.44-0.89)**
Previous back pain	0.82 (0.60-1.11)	1.38 (0.89-2.13)	2.56 (1.13-5.81)*

RR = rate ratio; OR = odds ratio; CI = confidence interval;*p < 0.05; **p < 0.01;

^a^RRs and ORs are adjusted for age, sex, BMI, smoking, alcohol consumption, SES, disease (self report of diagnosis), past sickness absence, and all other variables in the table.

^b^Poisson regression.

^c^Logistic regression.

### Prospective impact of psychosocial factors and previous back pain on self assessed prognosis of poor work ability

In Model I adjusted for age and sex, several factors emerged as predictors of poor self assessed work ability (Table 6): occupation (manual vs. non manual), high BMI, past sickness absence, previous back pain, high job strain, low decision latitude, low self-esteem, low coping, low sense of coherence, and low perceived control.

**Table 6:** Prospective impact of psychosocial characteristics and previous back pain at baseline on self assessed "poor" work ability.

Characteristic	Age and sex adjusted **(Model I)**^**†**^			Adjusted for the final model **(Model III)**^**‡**^		
		
	n	OR	95% CI	n	OR	95% CI
Occupation (SES)						
Non-manual	185	1 [ref]	.	.	.	.
Manual	112	*2.56*	*1.35-4.87*	.	.	.
Self-employed	40	1.27	0.44-3.68	.	.	.
Smoking, current	332	1.46	0.73-2.90	.	.	.
BMI	340	*1.11*	*1.04-1.20*	.	.	.
Alcohol consumption						
Low^a^	264	1 [ref]	.	.	.	.
Medium^b^	36	0.91	0.33-2.55	.	.	.
High^c^	25	2.33	0.84-6.51	.	.	.
Disease (self rep.diagnosis)	341	1.09	0.61-1.96	.	.	.
Past sickness absence	340	*2.37*	*1.28-4.40*	.	.	.
I Work environment						
Job strain	324	*14.85*	*2.54-86.69*	298	*11.71*	*1.69-81.01*
Psychological demands	327	1.07	0.95-1.21	299	1.07	0.93-1.22
Decision latitude	338	*0.84*	*0.75-0.94*	309	*0.83*	*0.73-0.95*
Social support at work	319	0.88	0.76-1.02	289	0.88	0.75-1.04
Overcommitment	330	1.00	0.94-1.07	300	1.05	0.97-1.14
II Emotional support	338	1.28	0.90-1.81	307	1.28	0.89-1.86
III Psychological resources						
1. Sense of coherence	338	*0.96*	*0.93-0.99*	308	0.97	0.93-1.00
2. Mastery	331	*0.86*	*0.78-0.95*	300	*0.84*	*0.76-0.94*
3. Self-esteem	329	*0.90*	*0.85-0.96*	299	*0.91*	*0.85-0.98*
4. Perceived control	312	*0.95*	*0.91-0.99*	285	0.97	0.92-1.01
Previous back pain	330	*2.78*	*1.31-5.92*	301	*2.62*	*1.18-5.84*

The odds ratio in italics denotes significance (*p *< 0.05). (Result from Model II, see text). In model III odds ratios for confounders are not reported as separate regressions were run for each of the 10 psychosocial variables and previous back pain.

n = number of observations used; OR = odds ratio; CI = confidence interval.

^a^Low (< 80 g/week for women, < 110 g/week for men)

^b^Medium (between 80-139 g/week for women, 110-169 g/week for men)

^c^High (≥ 140 g/week for women, ≥ 170 g/week for men)

^†^Adjusted for age, sex; ^‡^Adjusted for age, sex, BMI, smoking, alcohol consumption, SES, disease (self report of diagnosis), and past sickness absence.

In Model II, previous back pain, high job strain, low decision latitude, low self-esteem, and low coping were predictors of poor self assessed work ability (data not shown). In Model III, adjusting for past sickness absence caused little change in most estimates (see Table 6).

The multivariate logistic regression analysis of derived components of factor analysis (Table 5) showed that previous back pain (OR 2.56; 1.13-5.81; *p *= 0.02), high emotional support (OR 1.58; 1.02-2.46; *p *= 0.04), and low psychological resources 0.62 (0.44-0.89; *p *= 0.008) were related to *poorer *self assessed work ability at follow up, while the impact of work environment on work ability was no longer statistically significant (OR 1.39; 0.98-1.98; *p *= 0.07).

## Discussion

The main finding of this study is that, in a general middle aged working population, high emotional support was related to more sickness absence and also to poorer self assessed prognosis of work ability. Another finding was that low psychological resources and previous back pain were related to a poorer self-assessed prognosis of work ability.

### Determinants of sickness absence

A high level of emotional support was associated with both a higher frequency and longer duration of sickness absence. Such an association could be surprising since social support is considered protective against the development of depression in those exposed to life events [44]. Prospective studies, which control for baseline health status, consistently show increased risk of death among persons having few social relationships [45]. The association between high emotional support and increased risk of sickness absence is not surprising if such absence is seen as the effect of an "illness behaviour" rather than illness itself. High level of confiding/emotional support may encourage empowerment, security, and perceptions of control, which legitimize taking leave from work when ill [23]. Our findings confirm and extend previous findings in sickness absence, where high levels of confiding/emotional support were associated with higher frequency of short-term and long-term sickness absence [22]. Notably the definition of long-term sickness absence is >7 days in the Whitehall II cohort and >2 weeks in our study. Both definitions are in correspondence with the social insurance system in the two countries.

Findings of an association between increased job strain and more periods of sickness absence and days when adjusted for age and sex is in line with earlier research. The component of the demand-control model associated with lower sickness absence was "high decision latitude". This confirms the result of previous research, in particular that decision latitude [14,18] appeared to be a more important risk factors for sickness absence, than psychological demands and social support at work [19]. The association between job strain and sickness absence was reduced and was no longer statistically significant in the model adjusted for health behaviour and SES. The finding that previous back pain was related to the duration of sickness absence when adjusted for age and sex is consistent with previous research [46,47]. Poor psychosocial work conditions and physical workload are important risk factors for musculoskeletal pain [48]. The association between previous back pain and duration of sickness absence was no longer statistically significant in the model adjusted for health behaviour and SES. In both cases loss of effects after adjustment for SES can be an effect of over adjustment because of the strong relation between occupation, back pain and psychosocial factors at workplace [10,15].

### Determinants of self assessed prognosis of work ability

Knowledge of predictors for work ability is important for disability prevention, since work ability is an important predictor of duration of sickness absence [49], and return to work [50,51].

In this study work ability was related to a broader array of determinants than sickness absence. Work ability is the self-perceived relation between work demands and individual resources, defined as health and functional ability, education and competence, values and attitudes [52,53]. The association of psychosocial factors at work (high job strain and low decision latitude) and poor self assessed prognosis of work ability in the model adjusted for age, sex, lifestyle factors, SES, disease and past sickness absence (Table 6) is consistent with previous research [54,55]. However in the multivariate logistic regression adjusted additionally for previous back pain, emotional support and psychological resources (Table 5), the association between psychosocial work environment and poor work ability was no longer statistically significant.

Previous back pain was related to reduced self assessed prognosis of work ability in all models proposed (Tables 5 and 6). Back disorders constitute one of the most common causes behind long-term sickness absence and disability pension in Sweden [46,47]. Persistent musculoskeletal pain has been shown to be a predictor of reduced work ability [56]. If activity aggravates the pain (such as with heavy physical work load), and the individual avoids or reduces his activities, then pain may lead to disability. Cognitive function, and overall health were related to work ability in patients with chronic musculoskeletal pain [57].

Low individual psychological resources (coping and self-esteem) were related with self assessed prognosis of poor work ability (Tables 5 and 6). Coping and self-esteem are closely related to self-efficacy [58]. Self-efficacy, which is defined as confidence in being able to carry out a set of defined activities [59], has been highlighted in the literature as playing an important role for work ability and in the process of returning to work [60,61].

Just as could be seen for measures of sickness absence, high emotional support was related to *poor *prognosis of work ability in the multivariate logistic regression in Table 5. In this analysis, work ability is adjusted for age, sex, lifestyle factors, SES, disease, past sickness absence, previous back pain, work environment and psychological resources. In the analysis presented in Table 6, which was only adjusted for age, sex, lifestyle factors, SES, disease and past sickness absence, emotional support was not related to work ability. The results presented in Table 5 suggest that availability of emotional support provided by a person close's community outside work (family, friends, acquaintances), increases self-appraisal and boosts self-esteem, encourages to be absent from work.

A further question is how this applies to the perception of future work ability. It is possible to expect that absence in response to e.g. perceived strain at work, would actually reduce the risk of future inability to work. It is also possible that there is an element of reverse causality: that workers with a perception of decreased work ability may elicit more emotional support. This should be investigated further.

### Methodological considerations

Several studies have shown that self-reported sickness absence is highly correlated with administrative information on such absence and have concluded that self-reported data are sufficiently valid measures for its correct assessment [62,63]. Furthermore, self-reported data provide information on the entire period of sickness absence, including also the first week of sickness absence, when no sickness absence certificate is needed.

In the analysis of psychosocial resources and previous back pain, all multivariate analyses were adjusted for SES (socioeconomic status measured as occupation), as SES might cause both workplace exposures and poor health [64]. The model that includes SES might be over-adjusted because of the strong relation between occupation and psychosocial factors in the workplace [10,15]. The true relation between job strain, previous back pain, and sickness absence is probably between the unadjusted (Model I), and adjusted rate ratios (Model III).

The strength of the LSH study is its longitudinal design and a randomised sampling strategy. Several known and potential determinants, such as smoking, alcohol consumption, high BMI, and health status at baseline [10,54,65,66], were controlled in the analysis, which therefore limited confounding bias. Health behaviour (i.e. smoking) may be part of the causal pathway linking exposures to psychosocial factors at work and sickness absence and adjustment for these factors might reduce the true effect of the psychosocial work environment on sickness absence. The analysis was adjusted for past sickness absence, as previous research has shown that sickness absence is a strong predictor of future absence [29].

A limitation was the relatively small size of the study population and the subsequent low statistical power, leading to a possible non identification of true effects. Another limitation resulted from loss of a number of participants because of non-response and because some individuals were no longer gainfully employed at follow-up. There was a pattern of non-response correlated with occupation that tended towards a healthy worker effect selection, again leading to a possible underestimation of true effects. The baseline work ability data is not available and thus it was not possible to study the association between work ability at baseline and work ability at follow-up. The potential limits of self reported measures in terms of common method variance or shared response biases may lead to an overestimation of associations between exposure and outcome variables. Negative affect could be mediating the effect of personality on absenteeism [67] and could affect the response, but this was not controlled for in the model. A final limitation is that these data do not provide information about how the exposure variables (work environment, emotional support, psychological resources, back/neck pain) developed between baseline and follow-up

## Conclusions

In a general middle aged working population high emotional support was related to more sickness absence and also poorer self assessed prognosis of work ability. Our findings suggest that both sickness absence and work ability are dependent of life outside work and can be affected by a person's close community (relatives, acquaintances, and friends).

## Competing interests

The authors declare that they have no competing interests.

## Authors' contributions

NK, ES, and MK have made substantial contributions to the conception and design of this study and have contributed in the analysis and interpretation of data. NK has compiled data, produced tables, and has contributed to the statistical analysis. All authors have been involved in drafting the manuscript or revising it critically, and have read and approved its final version agreeing that it should be submitted for publication.

## Pre-publication history

The pre-publication history for this paper can be accessed here:

http://www.biomedcentral.com/1471-2458/10/648/prepub
